# Feature Point Descriptors: Infrared and Visible Spectra

**DOI:** 10.3390/s140203690

**Published:** 2014-02-21

**Authors:** Pablo Ricaurte, Carmen Chilán, Cristhian A. Aguilera-Carrasco, Boris X. Vintimilla, Angel D. Sappa

**Affiliations:** 1 CIDIS-FIEC, Escuela Superior Politécnica del Litoral (ESPOL), Campus Gustavo Galindo, Km 30.5 vía Perimetral, P.O. Box 09-01-5863, Guayaquil, Ecuador; E-Mails: paricaur@espol.edu.ec (P.R.); cchilan@espol.edu.ec (C.C.); boris.vintimilla@espol.edu.ec (B.X.V.); 2 Computer Science Department, Universitat Autònoma de Barcelona, Campus UAB, 08193 Bellaterra, Barcelona, Spain; E-Mail: caguilera@cvc.uab.es; 3 Computer Vision Center, Edifici O, Campus UAB, 08193 Bellaterra, Barcelona, Spain

**Keywords:** cross-spectral imaging, feature point descriptors

## Abstract

This manuscript evaluates the behavior of classical feature point descriptors when they are used in images from long-wave infrared spectral band and compare them with the results obtained in the visible spectrum. Robustness to changes in rotation, scaling, blur, and additive noise are analyzed using a state of the art framework. Experimental results using a cross-spectral outdoor image data set are presented and conclusions from these experiments are given.

## 1. Introduction

Recent advances in imaging technologies have increased the usage of cameras working at different spectral bands. As a result, novel solutions to classical problems have been proposed improving the results that can be obtained when only the visible spectrum images are considered (e.g., [1,2]). Infrared imaging represents one of the examples of such novel technologies. These images cover the spectral band from 0.75 μm to 15 μm, which is split up into the following categories: Near-Infrared (NIR: 0.75–1.4 μm), Short-Wave Infrared (SWIR: 1.4–3 μm), Mid-Wave Infrared (MWIR: 3–8 μm) or Long-Wave Infrared (LWIR: 8–15 μm). Images from each one of these categories have a particular advantage for a given application; for instance, NIR images are generally used in gaze detection and eye tracking applications [3]; the SWIR spectral band has shown its usage in heavy fog environments [4]; MWIR is generally used to detect temperatures somehow above body temperature in military applications; finally, LWIR images have been used in video surveillance and driver assistance (e.g., [5,6]). Recently, a personal thermal imaging device has been developed (FLIR ONE (http://www.flir.com/flirone/)) to be used with smartphones for applications such as security, home repairs, and outdoor activities. The current work is focused on the LWIR domain, which corresponds to the infrared spectral band farthest from the visible spectrum.

Like in visible spectrum image processing, different algorithms must be envisaged to handle images from the infrared domain (e.g., [7–9]). Actually, in order to tackle the applications mentioned above we have to address classical computer vision problems such as feature selection and tracking, image registration, pattern recognition, just to mention a few. The easiest way is to adopt classical tools from the visible spectrum to this new domain. One of these tools is the feature point detection and description, which has been a very active research topic during the last decade in the computer vision community. Feature detection and description in the LWIR spectral band is especially attractive in motion related applications, where lighting conditions are prone to change more rapidly than temperature (e.g., SLAM [10], egomotion [11], remote sensing [12]). Due to the large amount of contributions on this topic there were several works on the literature evaluating and comparing their performance in the visible spectrum case (e.g., [13–16]). However, to the best of our knowledge, there are no studies in the literature considering other spectral bands.

The current work proposes to study the performance of feature point descriptors when they are used in the far infrared domain (LWIR), and at the same time compare the results with those obtained in the visible domain (VS). The evaluation is performed using a data set from a cross-spectral stereo rig; hence a similar image is used to evaluate the performance in the two domains. Since there is a large amount of algorithms in the literature, we decided to select the most representative and recent ones. Hence, our study includes: SIFT [17], SURF [18], ORB [19], BRISK [20], BRIEF [21] and FREAK [22]. Although each descriptor has its own advantages and disadvantages, coarsely speaking they can be classified into two categories: (i) those based on image derivatives (e.g., SIFT, SURF) and (ii) those based on image intensities (e.g., ORB, BRISK, BRIEF, FREAK). Since images from the LWIR spectrum have less texture than those from the VS spectrum a lower number of features will be detected in the LWIR domain. However, it is difficult to predict whether this lack of texture would affect the performance of the different approaches when used with LWIR images.

The remainder of the paper is organized as follows: the evaluation methodology used for studying the performance in both spectral bands is presented in Section 2. Experimental results on a cross-spectral data set are presented in Section 3. Finally, conclusions and discussions are given in Section 4.

## 2. Evaluation Framework

The performance of different descriptors has been evaluated using the framework proposed by Khvedchenia [23]. This framework has been proposed for evaluating the performance of feature descriptors in the visible spectrum. It is intended to find the best approach for the correspondence problem when common image transformations are considered: rotation in the image plane, changes in the image size, blur and presence of noise in the images. In order to take into account all these possible changes, the given images are modified; then the different descriptors are applied and the matching with those points in the given images are considered as a ground truth. A brute force strategy is used for finding the matching, together with a L2 norm or Hamming distance, as detailed in Table 1. The brute force matching finds the closest descriptor in the second set by trying all the possible combinations. The percentage of correct matches between the ground truth image and the modified one is used as a criterion for the evaluation (Section 4). The transformations applied to the given images are detailed below:

**Rotation**: the study consists in evaluating the sensibility to rotations of the image. The rotations are in the image plane spanning the 360 degrees; a new image is obtained every 10 degrees.
**Scale**: the size of the given image is changed and the repeatability of a given descriptor is evaluated. The original image is scaled in between 0.2 to 2 times its size with a step of 0.1 per test. Pixels of scaled images are obtained through a linear interpolation.
**Blur**: the robustness with respect to blur is evaluated. It consists of a Gaussian filter iteratively applied over the given image. At each iteration the size of the kernel filter (K × K) used to blur the image is update as follows: K = 2*n* + 1, where *n* = {1, 2,…, 9}.
**Noise**: this final study consists in adding noise to the original image. This process is implemented by adding to the original image a personalized image. The value of the pixels of the personalized image are randomly obtained following a uniform distribution with μ = 0 and σ = *t*, where *t* = {0, 10, 20, …, 100}.

**Figure 1.**
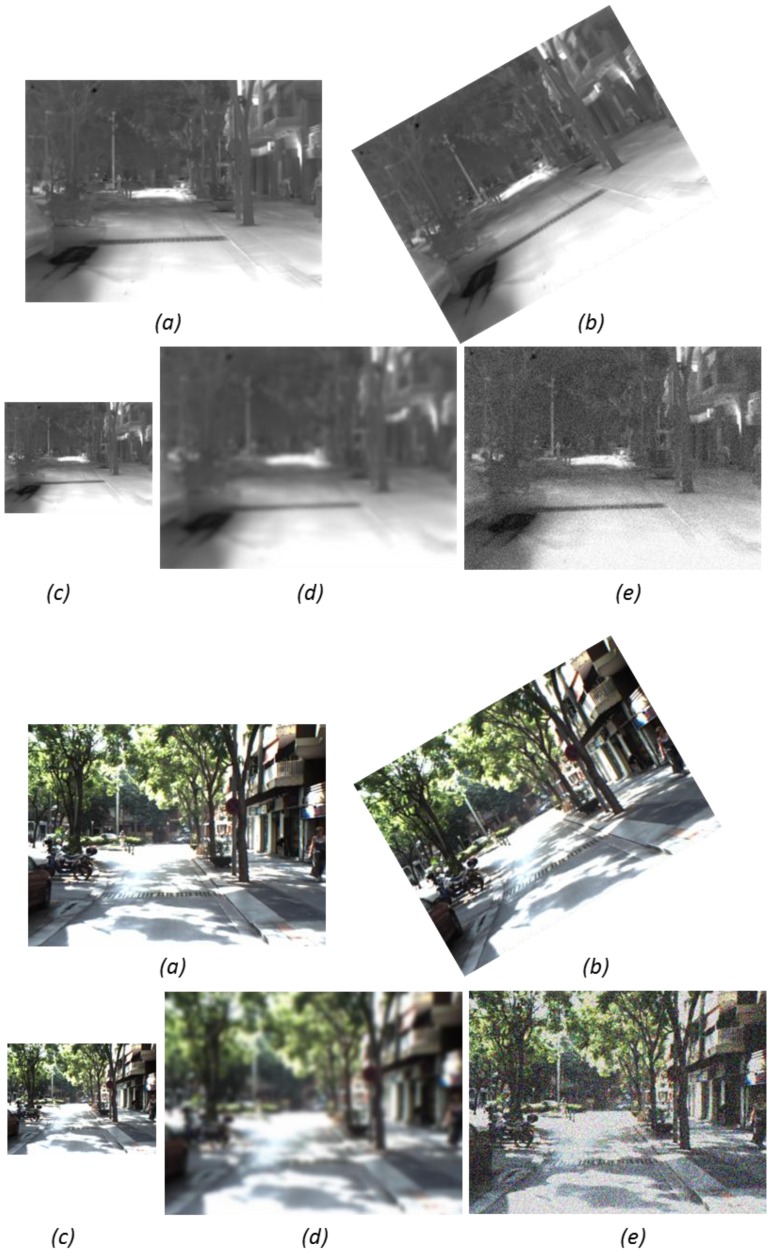
Illustration of a pair of images from the evaluation dataset ((**top**) LWIR and (**bottom**) VS) together with their corresponding transformed images: (**a**) original ones; (**b**) rotation; (**c**) scale; (**d**) blur; (**e**) noise.

In the original framework proposed by Khvedchenia, lighting changes were also considered, since that study was only intended for images in the visible spectrum. In the current work, since images from the LWIR spectrum are considered, changes in the intensity values won't follow the same behavior all through the image (like lighting changes in the visible spectrum). Intensity values in LWIR images are related with the material of the objects in the scene. In summary, a study similar to the lighting changes is not considered in the current work. Figure 1 shows an illustration of a couple of cross-spectral images (visible spectrum: VS and long-wave Infrared: LWIR images) together with their corresponding transformed images. The current work does not include comparisons on the execution time performance since execution time is an intrinsic characteristic of the descriptors; hence, independently of the spectral band the same performance will be obtained. Evaluations of the execution time performance for the different descriptors can be found in [23].

## 3. Experimental Results

The framework presented above has been used to evaluate the performance of different feature descriptor algorithms in a cross-spectral data set consisting of 40 outdoor images (VS and LWIR). The images were obtained with a multispectral stereo head consisting of a pair of cameras working in different spectral bands. The VS images were obtained with an ACE camera, from Basler, with a resolution of 658 × 492 pixels; while the LWIR images were obtained with a Gobi-640-GigE camera, from Xenixs. Both cameras are synchronized using an external trigger. Camera focal lengths were set so that pixels in both images contain similar amount of information from the given scene. This particular set up allows us to have images from different spectral bands of the same scenario. Note that the only preprocessing applied to the cross-spectral images is the color conversion of VS images to grey levels; there is no additional preprocessing or enhancement to highlight features or increase contrast.

**Figure 2.**
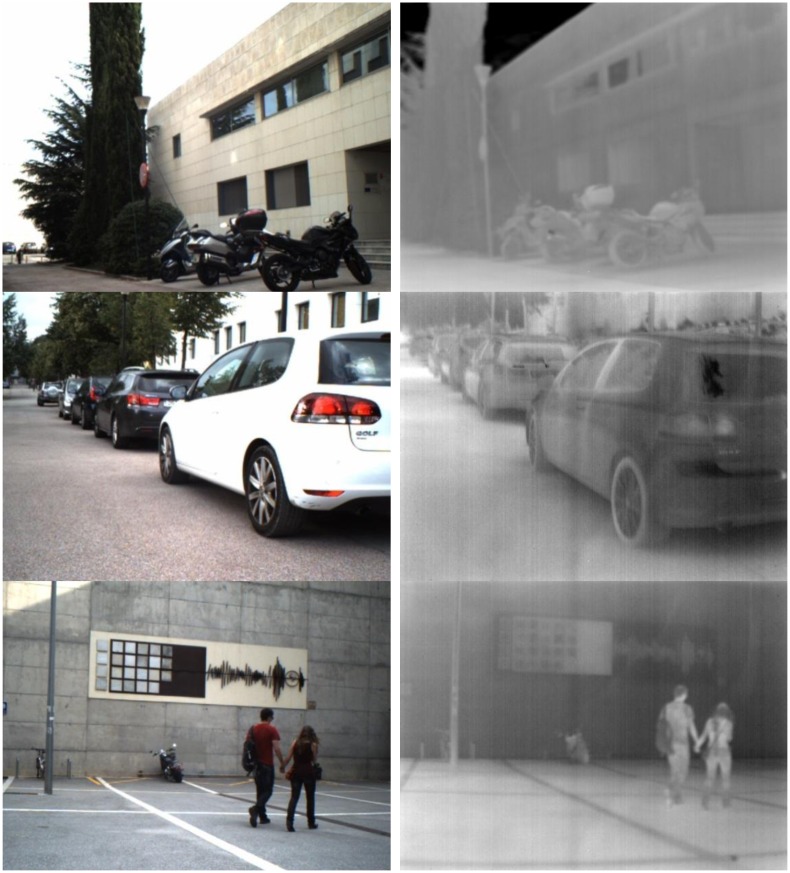
Pairs of cross-spectral images contained in the data set.

There are some recent works on the infrared image modeling and filtering (e.g., [24,25]) but this kind of study is out of the scope of current paper. Figure 2 presents some of the cross-spectral images contained in the dataset (http://www.cvc.uab.es/adas/projects/simeve/).

For each algorithm and transformation the number of correct matches, with respect to those in the original image, is computed and used for measuring the performance. In order to take into account the amount of points correctly detected by each of the tested algorithms, the results from SIFT are used as a reference. This allows us to measure the performance in each of the test and at the same time to compare the results with those obtained by other approaches. The proposed performance measure is computed as follows:

(1) $$\text{performance}=\frac{\#\text{correct}\;\text{matches}(\text{Alg}.i,\text{Transf}.j)}{\#\text{correspondences}(\text{SIFT},\text{Given}\;\text{image})}$$

Note that this performance measure can give values higher than one, which means that the evaluated algorithm obtains more features than those computed by SIFT in the given image. The algorithms evaluated in the current work are presented in Table 1. In the cases of BRIEF and FREAK the SURF algorithm is used as a detector. In ORB, BRISK, BRIEF and FREAK the Hamming distance is used, instead of L2 norm, for speeding up the matching. For each transformation (Section 2) a set of images is obtained; for instance, in the rotation case 36 images are evaluated.

**Figure 3.**
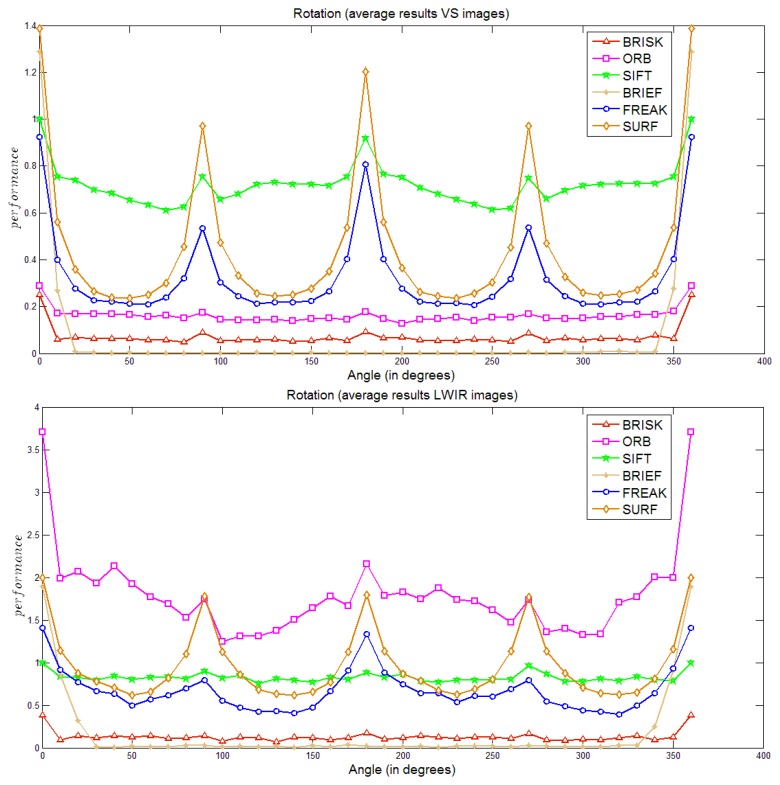
Performance in the rotation case: (**a**) visible spectrum; (**b**) LWIR spectrum.

Figure 3 depicts average results obtained when the given images are rotated between 0 and 360 degrees. It can be observed that in both cases (VS and LWIR) the most robust algorithm is SIFT. It can be appreciated that its performance remains almost constant along the different rotations (in particular in the LWIR case); it only decreases at the beginning (±10 degrees) but then does not change so much. On the other hand, the BRIEF algorithm (using SURF as a detector) is the most sensitive to rotations; actually, its performance drop to zero after applying a rotation of just 20 degrees in the VS case and after a rotation of 30 degrees in the LWIR case. In the case of SURF and FREAK, a slightly better performance was appreciated in the LWIR case where the performance does not decrease as much as in the VS case. Using the number of points detected by SIFT as a reference allows us to visualize that ORB has a considerably larger amount of points when used in the LWIR case. In spite of its performance is not as good as in the VS case, showing a large decrease just after a rotation of 10 degrees. Finally, BRISK shows a poor performance in both domains.

**Figure 4.**
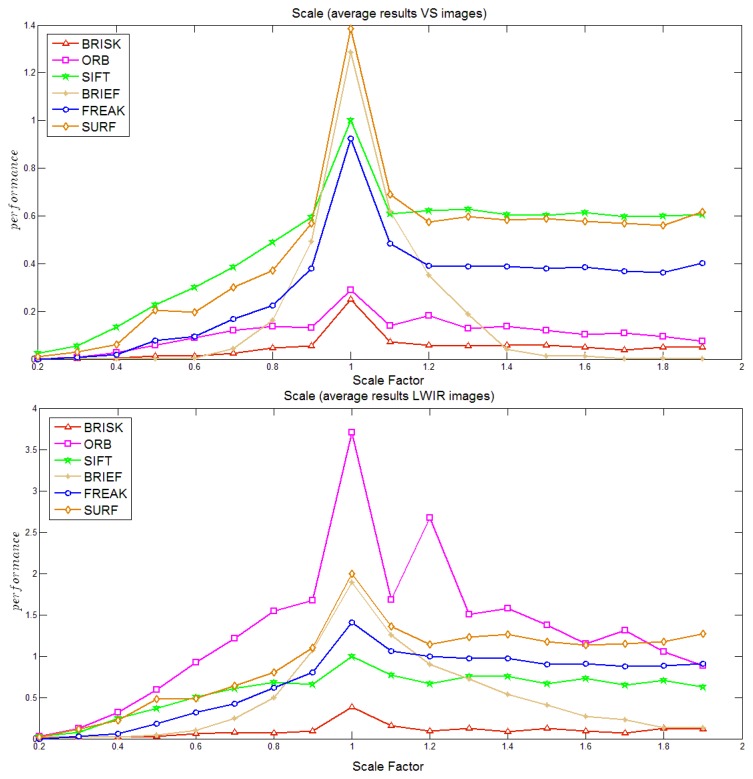
Performance to changes in scale: (**a**) visible spectrum; (**b**) LWIR spectrum.

In the scale study, on average the algorithms have a better performance in the LWIR domain than in the VS one. In both cases BRISK shows the worst performance followed by BRIEF. The algorithms SIFT, FREAK and SURF are the most stable with respect to scale changes. Similarly to in the previous case ORB is able to detect a large number of points in the LWIR spectrum. Even though its performance decay considerably, most of the times is the algorithm with most detected points. On the contrary, it is among the algorithm with less detected points in the VS domain. Its performance in the VS domain is quite stable. Figure 4 shows these results.

**Figure 5.**
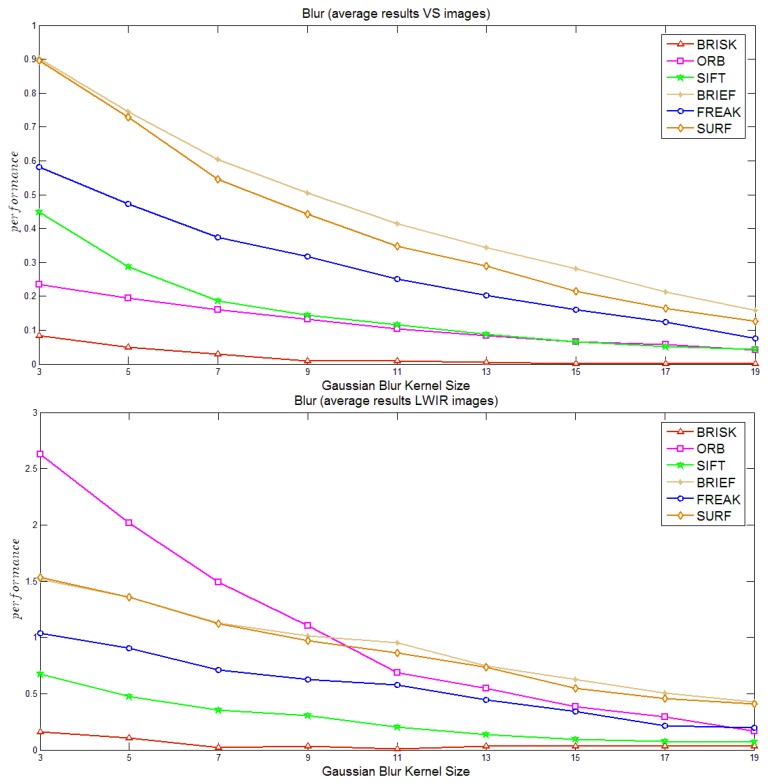
Performance to image degradation (blur): (**a**) visible spectrum; (**b**) LWIR spectrum.

Figure 5 presents the study of robustness of the different algorithms when the given images are degraded using a Gaussian filter of increasing size. In general all the algorithms in both spectrums are equally affected showing a decrease in performance with the increase of kernel size. In the particular case of LWIR, ORB shows the worst performance; in other words it seems to be the most sensitive to blur. This fact can be appreciated in the fast decrease in performance.

**Figure 6.**
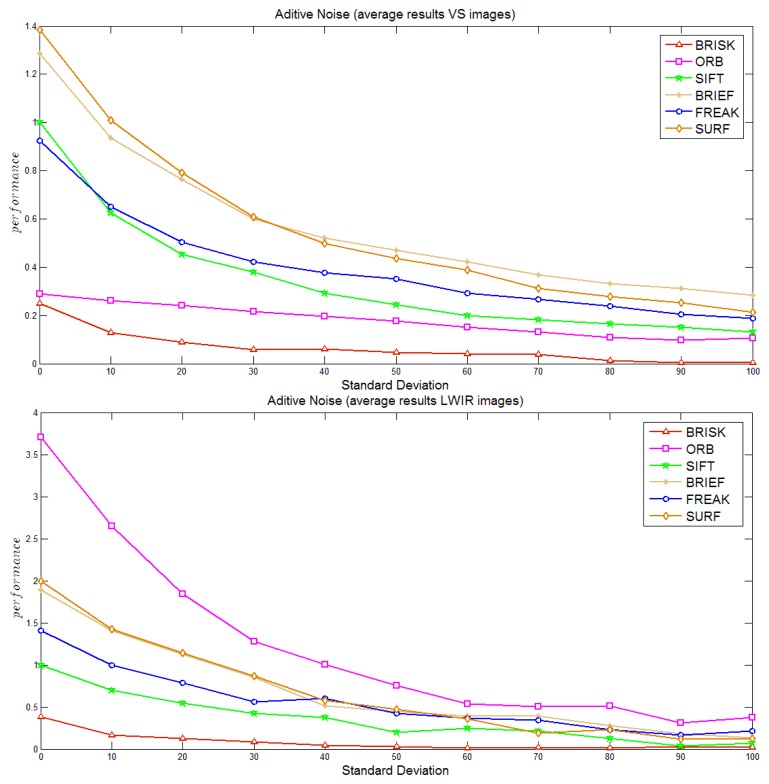
Noise case study: (**a**) visible spectrum; (**b**) LWIR spectrum.

Finally, Figure 6 shows the curves obtained when additive noise is considered. As expected the performance of all the algorithms is degraded with noise. Similarly to in the case of blur, the performance of all the algorithms decreases with a similar behavior. In the VS spectrum ORB is one of the most robust algorithms, while its performance in the LWIR is this worst. This bad performance and sensitivity to noisy data can be explained by the nature of images (low contrast) together with the way this algorithm detect feature points (based on FAST, which uses an intensity threshold between the center pixel and those in a circular ring). On the contrary, in the VS domain, although ORB is affected by noisy data like all the other algorithms, it is not as evident in the LWIR case because of greater contrast noise in the images.

## 4. Discussion

As mentioned in Section 1, the descriptors considered in the current work can be coarsely classified as: (*i*) those based on gradient information; (*ii*) those based on intensity information. We study whether is possible to find some correlation between the descriptor's family and the improvement or the drop in performance in the different experiments. The lack of texture in the LWIR domain was one of the focuses of our study. Since it is one of the characteristics of LWIR images we tried to see how it affects the performance mainly on those descriptors based on the usage of gradient information. As mentioned above, the images used to study the algorithm performance (LWIR and VS) are the ones provided by the cameras. There is no a preprocessing to filter or improve their contrast.

Looking at the results presented in the previous plots we can conclude that the algorithm ORB is the one that detects most of the features in the LWIR domain. This conclusion is related with the lack of texture and low contrast of LWIR images that affects those algorithms based on gradient information. In order to unveil additional conclusions we propose to compute the recall, similar to [14], for each experiment with the different transformation. It is computed as follows:

(2) $$\text{recall}=\frac{\#\text{correct}\;\text{matches}}{\#\text{correspondences}}$$

where #*correspondences* represents the number of features detected/described in the given image by the algorithm being tested (used as a ground truth), and #*correct matches* are the matches obtained after transforming the image and detecting/describing feature points with the algorithm being tested. This recall is computed for the different combinations of algorithms and transformations ( ${\text{recall}}_{i}^{j}$, where *i* = {blur, rotation, noise, scale} and *j* = {BRISK, ORB, SIFT, BRIEF, FREAK, SURF}) and for every set of images ( ${\text{recall}\_\text{LWIR}}_{i}^{j}$, ${\text{recall}\_\text{VS}}_{i}^{j}$). Finally, we propose to compute the average of differences between ( ${\text{recall}\_\text{LWIR}}_{i}^{j}$, ${\text{recall}\_\mathrm{VS}}_{i}^{j}$); this will be referred to as *ARD: Average Recall Difference*. This value can be used to compare the performance of an algorithm in each spectral band (a negative value means its performance is better in the visible spectrum than in the infrared one):

(3) $${\text{ARD}}_{i}^{j}=\frac{{\text{∑}}_{k=1}^{n}{\text{recall}\_\text{LWIR}}_{i_{k}}^{j}-{\text{recall}\_\text{VS}}_{i_{k}}^{j}}{n}$$

where *n* depends on the transformation, for instance in the rotation case it consists of 36 elements (see Figures 3, 4, 5–6 for more details). These *ARDs* are presented in Table 2; since the pairs of cross-spectral images contained in the data set correspond to the same scenario, the *ARD* gives an idea of the difference in performance for each transformation. Since this study is focused on the LWIR spectrum we identify the algorithms with best behavior in this domain. On average, the algorithm SURF has the best behavior in the LWIR in the blur transformation; while in the case of rotation SIFT seems to be the best one, which somehow corresponds with the results presented in Figure 3b where SIFT is the most stable one (it was also mentioned on page 6). In the case of noise all the algorithms have a bad performance in the LWIR spectrum, being BRISK the less sensitive one. Finally, in the case of changes in scale the algorithm SIFT has a better behavior in LWIR domain than in VS, this can also be noted by comparing curves in Figure 4, where SIFT (followed by FREAK) shows a quite robust behavior with respect to changes in scale in the LWIR spectrum.

## 5. Conclusions

This work presents an empirical evaluation of the performance of the state of the art descriptors when they are used in the LWIR domain and compared against the results from those obtained in the visible spectrum. Although it is difficult to make a conclusion about which is the best feature detector and descriptor algorithm, since it would depend on different factors and according to the Table 2 there is not a clear winner, we can say that SIFT is among the best ones, showing good performance in most of the experiments. As a future work we will explore the usage of image preprocessing to enhance LWIR before feature detection and description. Due to the nature of LWIR images, and according with recent works, it seems that results could be improved with some image preprocessing.

## Figures and Tables

**Table 1.** Algorithms evaluated in the study.

**Feature Descriptor Algorithm**	**Matcher Norm Type**
SIFT	L2 Norm
SURF	L2 Norm
ORB	Hamming Distance
BRISK	Hamming Distance
BRIEF (SURF as a detector)	Hamming Distance
FREAK (SURF as a detector)	Hamming Distance

**Table 2.** Average Recall Difference for the algorithms evaluated with the framework presented in Section 3 (bold values correspond to the algorithm that has the best relative performance in LWIR for the tested transformation).

***ARD***	**BRISK**	**ORB**	**SIFT**	**BRIEF**	**FREAK**	**SURF**
Blur	0.0442	−0.1323	0.1064	0.1149	0.0904	0.1425
Rotation	0.0450	−0.0762	0.0726	0.0109	0.0584	0.0013
Noise	−0.0427	−0.2921	−0.0764	−0.1266	−0.1273	−0.1106
Scale	0.0598	−0.0250	0.1564	0.0853	0.1271	0.1126
